# Material characterization and selection for 3D-printed spine models

**DOI:** 10.1186/s41205-018-0032-9

**Published:** 2018-10-19

**Authors:** John Hao, Raj Nangunoori, Ying Ying Wu, Mabaran Rajaraman, Daniel Cook, Alex Yu, Boyle Cheng, Kenji Shimada

**Affiliations:** 1 Carnegie Mellon University, Carnegie Institute of Technology, CERLAB, 3000 Forbes Ave., Pittsburgh, PA 15213 USA; 2 Allegheny General Hospital, Department of Neurosurgery, 320 E North Ave., Pittsburgh, PA 15212 USA

**Keywords:** 3D-printing, 3D-print, Biomechanics, Bone, Cadaver, Materials, Mechanical test, Model, Neurosurgery, Orthopedics, RPM, Qualitative, Sawbone, Stereolithography

## Abstract

The two most popular models used in anatomical training for residents, clinicians, or surgeons are cadavers and sawbones. The former is extremely costly and difficult to attain due to cost, ethical implications, and availability, while the latter is said to not have the same tactile fidelity or mechanical properties as human bone. This study examined the potential use of 3D-printed phantoms to emulate cadaveric, human vertebrae, in hopes of acting as a future use over cadavers. In so doing, we developed 3D-printed MedPhantom®, with the intended use to offer similar tactile feel, mechanical characteristics, and visual appearance as human bone. In order to quantify tactility, a mechanical test was developed where a 5-mm diameter diamond-coated bur spinning at 75,000 RPM swept across the specimens while continuously recording the resultant forces (N) and moments (N-cm), The bur sweep motion is common in orthopedic surgery and neurosurgery. Since most 3D-prints do not offer internal, trabecular structure similar to bone, an algorithm was written to create a stochastic framework of internal mesh to mimic cancellous bone within an STL (stereolithography) file. The ranges of mesh parameters were chosen after several visits with the neurosurgeons participating in the project. In order to quantify structural combinations of wall thickness, gap sizes, and varying cylindrical radii within a print, 1000 RPM compression test with a 5-mm diamond-coated bur was performed with resultant forces (N). Two sample t-test shows statistical significance that samples are not equal to the vertebrae (*p* < 0.05). Results from the bur sweep test showed 15% Gypsum® powder mixed with 100% Clear® Formlabs resin and 10% Castable® resin mixed with 90% Clear® resin were nearest to human, cadaveric vertebrae, with the difference of force and moment in the x-direction at only 5 N and 7–9 N-cm, respectively. Structural compression results showed that a 2 mm cortical wall, 4 mm or 5 mm gap size between cylinders inside the structure, and 0.25 mm radius of internal cylinders were the best fit parameters to match human vertebrae.

## Introduction

### Overview

THIS study aimed to characterize material and structure for use in CERLAB’s 3D-printed MedPhantom Spine, with the overarching goal of developing an alternative model and obviate the need for human cadaveric vertebrae and sawbones, reducing cost, saving on time, and quantifying tactile fidelity for surgeons outside of the operating room or surgical rehearsal. Currently, there is no mechanical or quantitative method to determine tactile feel in regard to burring or instrumenting bone, which is a characteristic and scale used by surgeons and clinicians when working with bone or tissue. In order to test the tactile similarity of our 3D-printed model to bone, we opted to compare the models to human vertebrae. Two different mechanical tests were done for the two different parameters being observed: a custom bur sweep test for material combination testing and a compression test for structural dimension combinations, both of which are explained in detail within the Methods section (II).

**Fig. 1:**
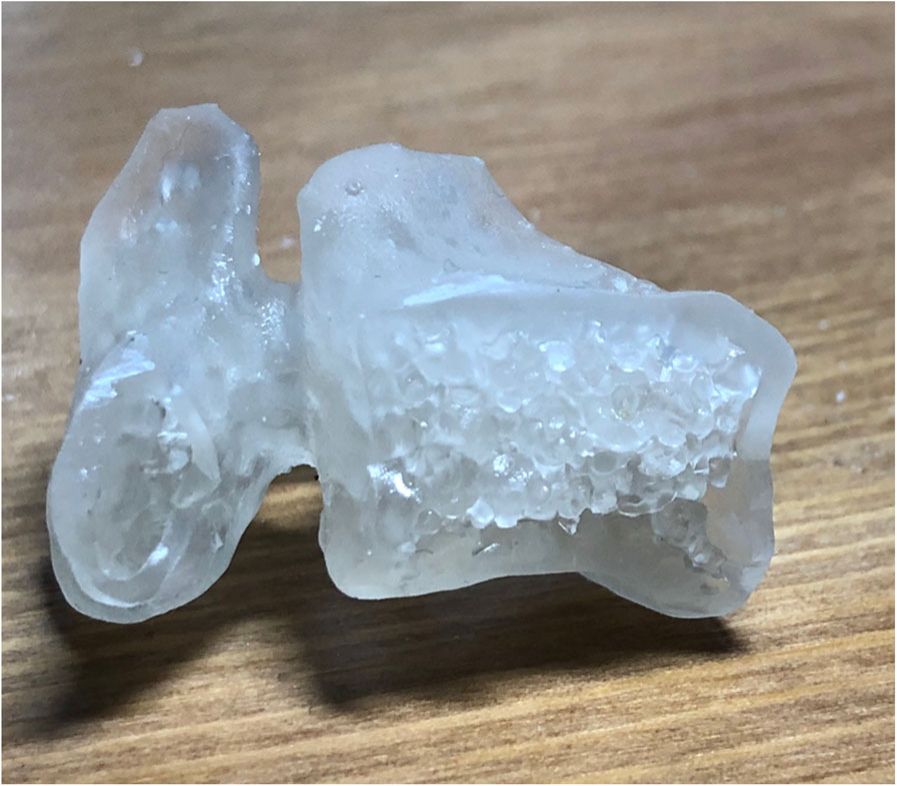
Section cut of 3DP vertebrae showing internal mesh trabecular structure while housed within a thick cortical wall, attempting to model real bone. Code was written to add a stochastic framework of a mesh within a thick wall while the model was printed with 100% Clear® resin off the Form 2® SLA Printer

One of the chief complaints by neurosurgeons and clinicians we spoke to throughout our partnership with AGH, was they felt current models are either too costly, difficult to attain due to cost, ethical implications (i.e. animal models), availability, or do not have the same tactile fidelity as human tissue and bone. It was imperative to obtain a model that has the same architectural morphology as bone with similar visual appearance but can also provide a suitable tactile and mechanical feedback similar to what human bone offers. However, that poses the problem of how one goes about quantifying or providing data to support an opinion that is tactile feel, an often-subjective parameter. (see Fig. 1).

**Fig. 2:**
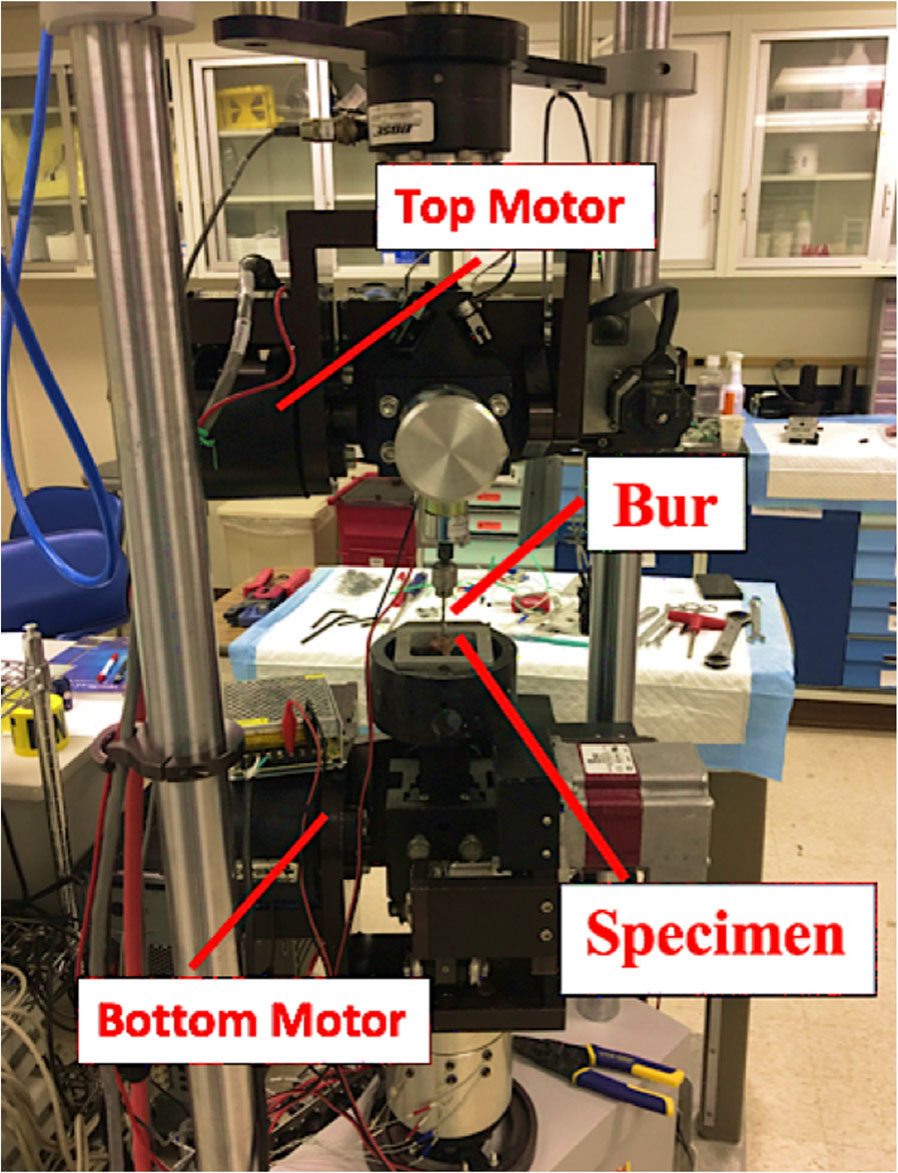
Setup of six-degrees-of-freedom machine. Similar to the freebody diagram in Fig. 4, the specimen is clamped in a cup within the bottom motor while the bur is attached to the top motor, sweeping in an arc motion across the top of the specimen. The motors are controlled pneumatically through a pressure system within the building. The increased pressure allows for various complex motions within the six-degrees-of-freedom (3 modes of translation, 3 modes of rotation), all with the capability of moving in sync with each other. Load cells, with a sensitivity of 0.01 N and max load of 10 kN, are located near *both the top and bottom motors*

**Fig. 3:**
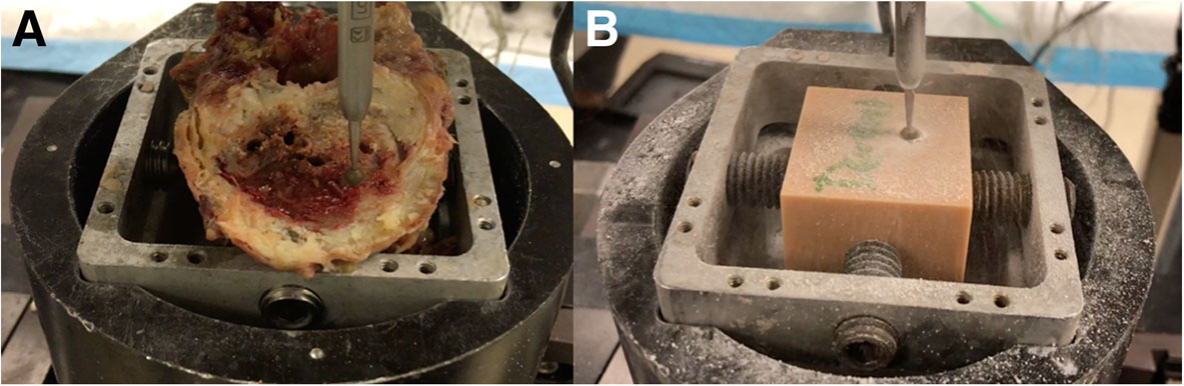
(**a**) Close-up image of bur in its holder attached at top motor sweeping across the specimen (human vertebrae) that is clamped within a cup on the bottom motor. The bur spun at 75,000 RPM which only required a foot on the pedal to run it, similar to how most burs operate in surgery. (**b**) Close-up image of bur sweeping ac*ross Dental® resin printed cube*

Therefore, we developed a mechanical test to mimic what occurs in orthopedic and neurosurgery and subsequently recorded resultant forces and moments from the test. In short, a drilling bur was attached to a fixated cup and swept across various combinations of 3D-prints, as well as human cadaveric spine to compare it to (see Figs. 2 and 3). This sweeping motion of the bur at speed as high as 75,000 RPM is commonplace in orthopedic and neurosurgery, or any surgery involving bone and tissue [1, 2]. For example, in a disc replacement surgery, a drilling bur would be initially used to shave off the vertebrae sandwiching the intervertebral disc prior to taking out the damaged disc and ultimately replacing it with a spacer filled with bone graft [3]. Apart from the human vertebrae attained from cadavers, 3D-printed material combinations were developed by experimenting with several light-sensitive photopolymer resins (Formlabs®) and powder mixture combinations (Gypsum powder and Kevlar fiber).

The second parameter tested was for internal structural combinations. Bone has three predominant layers arranged in a sandwich configuration: an outer hard cortical shell layer with a spongy, cancellous (trabecular) middle layer which houses osteoblasts and osteocytes and is also referred to as the bone marrow [4]. Trabecular bone, which lies between the cortical layers is said to have a spongy, mesh structure [5]. In order to model this structure, an algorithm was written on Python to add a random, stochastic framework of cylindrical lattices within the given print, in this case the 3DP specimens being tested. In short, this algorithm took an empty of shell of an existing STL file, then proceeded to add an outer shell and internal cylinders. Cylinders were allowed to overlap but only once otherwise this could lead to too large of a clump, which would not achieve the idea of a mesh. The three parameters the user could change were outer shell thickness which represented the cortical wall thickness, the radius of the cylinders and gap sizes present within the print which represented the trabecular, spongy bone. The 5-mm diamond-coated bur spinning at 1000 RPM going through axial compression then pierced completely through the printed specimens and human, cadaveric vertebrae. A full compression test through the entirety of the specimens allowed for a full mechanical characterization at each layer. A higher speed RPM during the bur-sweep test mimicked motion and speed during orthopedic surgery [6].

Although there are several types of 3D-printing methods, the ones used in this study was stereolithography (SLA) and fused deposition modeling (FDM). SLA uses a laser to cure a liquid photopolymer to solid from the bottom up which was done using Formlabs Form 2® printer [7]. FDM uses an extrusion process that extrudes melted plastic layer by layer onto a build plate [8]. All printed specimens were printed from the Form 2® SLA printer, while FDM prints were outsourced from Shapeways™. The FDM prints consisted of PLA (polyactic acid) unpolished, PLA polished, and nylon. Different gypsum powder and Kevlar pulp combinations mixed with Formlabs® resins were experimented with during the bur sweep testing (see section II for details). Gypsum powder is primarily used in agricultural and construction applications by hardening and setting the material to be used for casting and construction [9]. Kevlar is most popularly known to aid in the creation of the famous Kevlar vests, but this was an aramid pulp filled with highly fibrillated chopped fibers that are known to offer high strength, modulus and toughness [10].

The mechanical properties of the resins used from the Form 2® printer can be found on the Formlabs® website [11]. Although ultraviolet (UV) light curing at 405 nm is shown to improve mechanical characteristics at, we found that this also in turn makes the material more brittle, making the print fail easily under a high-load of drilling, which in our case is at 75,000 RPM using a diamond-coated bur tip [11]. While the ultimate tensile strength increases, the elongation at failure percentage decreases when UV-curing for Clear® resin, going from 12% to 6.2%, meaning it takes less elongation before the print fails, such as cracking or breakage [11]. Flexible® has elastomeric properties with an elongation at failure value at 60% pre-cure and 75–85% post-cure, and Tough® resin has an elongation failure of 42% pre-cure and 24% post-cure [11]. The higher the elongation to failure percentage is, the higher chance the print has to matching mechanical characteristics of bone without catastrophic failure due to brittleness. From this, we wanted to find an optimal cure time to improve mechanical characteristics where it increases hardness of the material to match that of human bone, but does not increase the brittleness, thus susceptibility to fail whether from a fracture, crack, or breakage. Therefore, the specimens were all UV-cured for only 10 min, as opposed to the recommended 30-min UV-curing time from Formlabs®.

Ultimately, the goal of this paper was to develop a model that can be used as a replacement for cadavers, specifically the vertebrae in the human spine. In order to do so, we developed and characterized 3D-printed material and structure and subsequently compared results to human, cadaveric vertebrae in order to find the best possible fit in regard to likeness. Finding a suitable 3D-printed model could potentially obviate the need for cadaveric specimens, which would reduce cost but also provide quantitative tactile fidelity which is integral in surgical rehearsal outside of the operating room.

### Previous work

Currently, surgeons and residents have limited options for rehearsal outside of the operating room. Available options include cadaveric specimens, animal models, or sawbones. Cadaveric specimens provide the most anatomic and tactile realism, but they are limited in availability and require costs as high as $2000–$4000 USD, as well as the cost of maintaining them upon the costly attainment [12]. In addition, the acquisition of cadavers for training courses is made difficult due to regulatory hurdles and the need for specialized storage facilities [13]. Ethical concerns often limit the ability for surgeons to practice on animal models, while sawbones is better for demonstrating anatomy without providing necessary tactile feedback or showing pathology that could translate to skills in the operating room. Sawbone models, solid rigid polyurethane foam, are commonly used to also test for ASTM standards, acting as one of the more popular models for human bone that is not a cadaver [14]. However, there is little to no differences in internal structure or cortical shell thicknesses like the 3DP models offered in this study. Furthermore, Zech et al. 2006 showed large differences and variations when comparing sawbones to bovine and human bone [15].

Although Stratasys® currently offers a wide range of 3D-printed anatomical models, they have a stronger focus in the field of pathology-specific anatomy or tissue emulation [16]. Their 3D-printed models are also used for vasculature due to the complex nature of humans’ vasculature systems, which require an intensive amount of preparation pre-operatively [16]. While Stratasys® claims their prints offer similar mechanical compliance to human tissue, there remains the issue of cost. Stratasys’s cost of 3DP anatomical models have said to be extremely costly compared to our current SLA printing from the Form 2® printers, where the former’s PolyJet® SLA resin prints can range from $6 K- $20 K (depending on size, orientation, material, and not including ownership of printer) while the latter’s Formlabs® SLA resin prints costs as low as $3500 (including ownership of printer and resin).

After further extensive research, there was little to be found for 3D-printed trabecular or internal framework within a solid print, specifically with the aim to mimic human cancellous or trabecular bone. One of the most important aspects of achieving tactile similarity to human bone for 3D-prints is to make certain there is an internal meshing to mimic spongy, trabecular bone, which is a facet this study attempted to produce and subsequently investigate.

## Methods

The first mechanical test performed in this study was done in order to address tactility, an oft-subjective parameter. The results from the tactile tests will later be compared to a separate qualitative study surveying and interviewing rotating surgeons, residents, and clinicians who will personally rate and scale the types of 3D-printed (3DP) models compared to bone. This will shed light on the contrast between the mechanical results from this study and qualitative surveys from surgeons and residents practicing in the Neurosurgery Department at Allegheny General Hospital (AGH). The second mechanical test investigated structure, with the specific intent of mimicking a cancellous to trabecular morphology that is seen in human bone.

### Material: Bur-sweep test

#### Resultant forces & moments

**Fig. 4:**
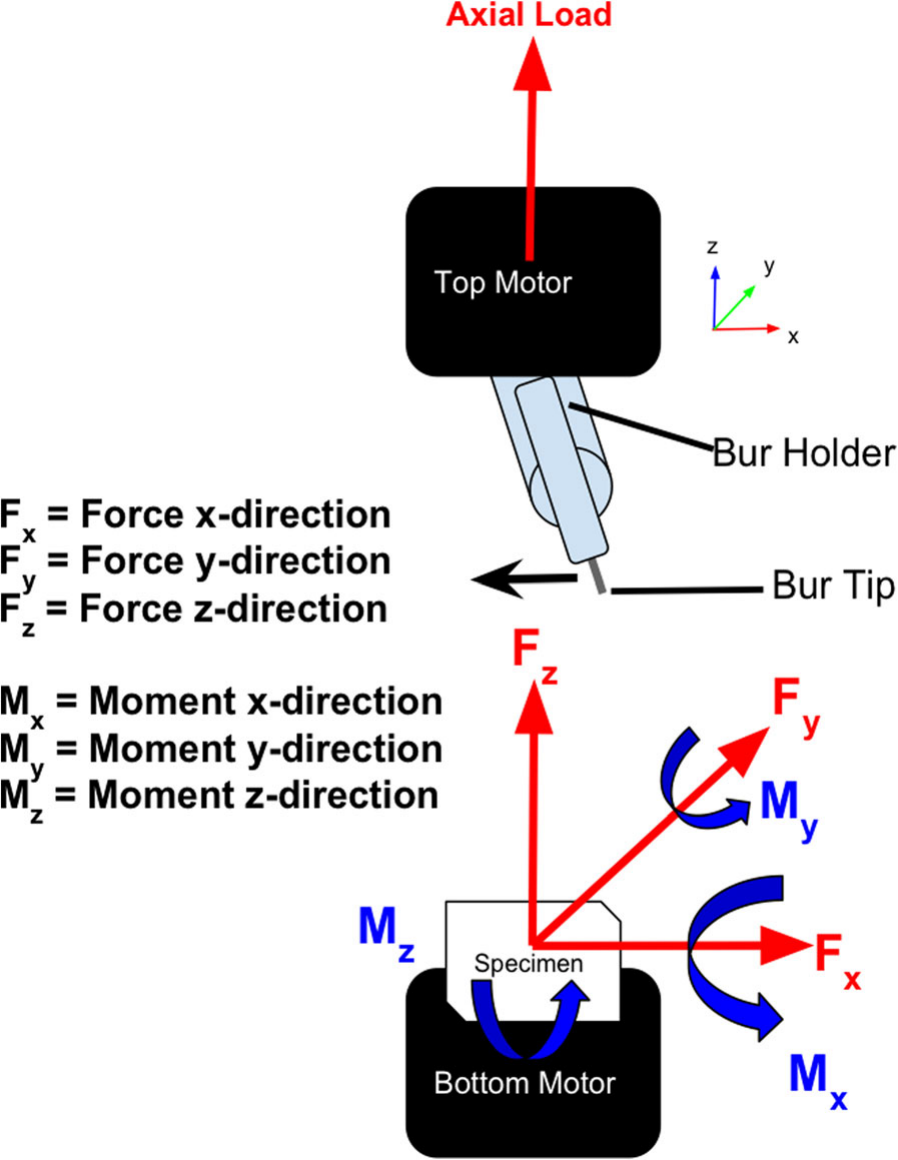
Red represents all force vectors while blue represents moments in counter-clockwise direction. F denotes force, M denotes moment, and x, y, and z denote the coordinate plane/direction. Motors are colored in black, bur and its 3DP holder in light blue, specimen in white supplanted within a cup held by bottom motor. Black arrow to the left of the bur tip indicates the direction the bur sweeps while the specimen remains immovable, clamped within a cup fixated on the bottom motor. Load cells are housed within the top and bottom motors

The type of motion being mimicked in this test is what occurs during surgery, where the surgeon takes the bur spinning at around 75,000 RPM and slowly glides it across the tissue, generally bone. The bur’s ultimate trajectory in this test followed that of a sweeping arc, where the resultant forces (N) and moments (N-cm) were recorded as the rotating bur glided across the plane of the specimen for the 3DP models and cadaveric human vertebrae, separately. The moment and the force were recorded in both the x and y-direction (see Fig. 4 for free body diagram). We were primarily concerned with the amount of change in an object’s motion (force in N) and the measure to cause a body to rotate about its axis (moment in N-cm).

The force and moment profiles across the transverse plane of the 3DP models and cadaveric models were investigated. Specifically, force in the x and y-directions (F_x_ and F_y_) and moments in the x and y-directions (M_x_ and M_y_) where the x and y subscripts denote coordinate plane (see Fig. 4). The load cells (manufactured by Bose™) for the top and bottom motors fed back the resultant moments and forces for all three coordinates (x, y, and z) (see Fig. 2 for setup).

#### Bur sweep setup

To perform the bur-sweeping motion, a custom six degrees-of-freedom (6DoF) machine located at Allegheny General Hospital was used (manufactured by Bose™). The 6DoF machine is primarily used for biomechanics testing involving range of motion tests of the spine, such as analyzing change in degrees of motion pre and post-instrumentation. The machine is hooked up to a pneumatic pressure system within the building, where a constant pressure is being fed into the machine to allow complex motions to be ilicited. The bur sweep method came about through development of a mechanical test to find the “best fit” for quantifying tactile feel. The bur was set up and housed in a 3DP case with all-threaded screws that clamped the bur. This was then subsequently attached to the top of the custom machine and held in place within a bolted cup (see Fig. 2). The specimens (3DP models and cadaveric models, separately) were then fixated within an open, rectangular metal cup, where threaded screws were then used from four sides to fixate the specimen; this was then held in place within another bolted cup on the bottom of the custom machine.

Since this machine is primarily used for spine in biomechanical testing, it is able to elicit complex motions such as lateral bending (side to side), flexion-extension (front and back) and axial torque (twisting). For our experiment, we required only the lateral bending motion with the bur “sweeping” in an arc motion, gliding along the transverse plane of the specimen. The bur speed was set at 75,000 RPM. Prior to every day of testing, a tare or calibration test needed to be done where the motors go through all six degrees motions (flexion-extension, lateral bending/side to side, and axial torsion/twisting) for a total of three cycles each while accompanying voltage values fed back from the data acquisition (DAQ) and are subsequently used to convert future voltage values obtained during testing into Newtons or Newton-centimeters. This only needs to be done once per day, not before every single test or specimen if they are being tested on the same day. The DAQ feeds back real-time, continuous data (time, axial displacement, distance, forces in x, y, z directions and moments in x, y, z directions) through two coupled load cells (manufactured by Bose®) on the top and bottom of the machine, as well as motion tracking from NDI Principles® if needed. The final output values were in time (seconds) and voltages which are tabulated in LabView® and then converted to Newtons, Newton-cm, and cm for the varying parameters observed using a custom-written Microsoft Excel® macro.

#### Bur sweep speed

**Fig. 5:**
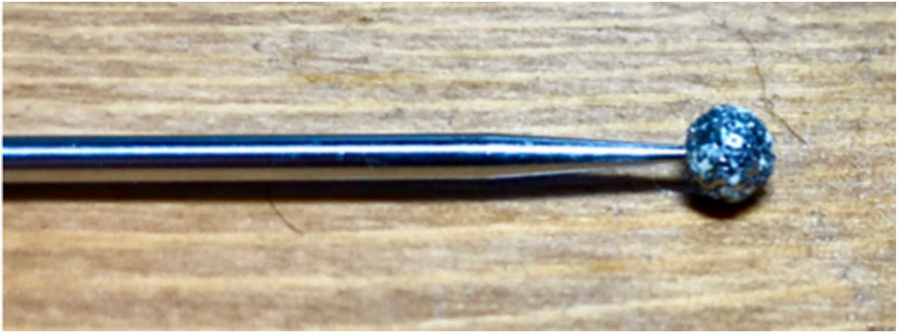
5-mm diameter diamond-coated bur tip used in all tests. This bur tip is common *in orthopedic and neurosurgery*

A 5-mm diamond-coated bur was used since this is a commonly used bur in the operating room by orthopedic and neurosurgeons (manufactured by Medtronic™) (see Fig. 5). The bur furthermore operated and spun at 75,000 RPM, which is a common speed used in orthopedic and neurosurgery, especially in that of spine [4, 1]. The rate of sweeping was at 1.5 degrees per second, beginning at one side of the pendulum arc then sweeping to the other; this speed was chosen to allow the machine ample time to feedback results with less noise, while going at a speed as close to surgeons’ movements during surgery without compromising DAQ feedback. The resultant moments (N-cm) and forces (N) were fed back from the load cell to the DAQ, which outputted initial values in voltages. These voltages were then converted by multiplying by the scale factor of the load cell, which can change based on the waveform of each test since tare and calibration values can slightly affect final force and moment values.

### Structure: Drill compression test

#### Drill compression setup

The portion of the drill compression test also used the six-degrees-of-freedom machine at AGH, however without the sweeping motion and just a straight downward compression. The machine’s settings were set at a feed rate of 1 mm/s, plotting a distance (cm) versus force (N) graph. The samples were fixated and clamped on the bottom while a custom 3D-printed holder was held at the top with a constantly 1000 RPM rotating 5-mm bur tip. Once the bur began spinning, the machine would begin to drop the bur tip down at 1 mm/s while the force and distance were calculated and fed back using the 10-kg load cell within the bottom motor (manufactured by Bose®).

#### Drill compression bur

The bur tip used for the structural compression test was again a 5-mm diamond tipped bur. The goal of the compression test was to determine whether the artificially engineered trabecular geometry could be identified between samples that had the mesh versus those that did not. Therefore, a lower speed (1000 RPM) was chosen for this test since prior pilot studies and tests using lower speeds have demonstrated improved noise-to-signal feedback.

### 3D-printed combinations

#### Material: Bur-sweep combinations

Prior to running the bur sweep tests on thoracic, cadaveric spines, we first began doing so on several 3DP combinations. All 3DP models measured 40 cm × 30 cm × 30 cm. The Form 2 printer from Formlabs is a SLA 3D-printing method and the Clear V4®, Tough TOTL-05®, Flexible V2®, and Castable V2® resins provided from Formlabs were used. Any prints requiring a mixture (both resin or powder) was done prior to pouring into the tank for printing and all prints were printed at 0.05 mm resolution.

**Fig. 6:**
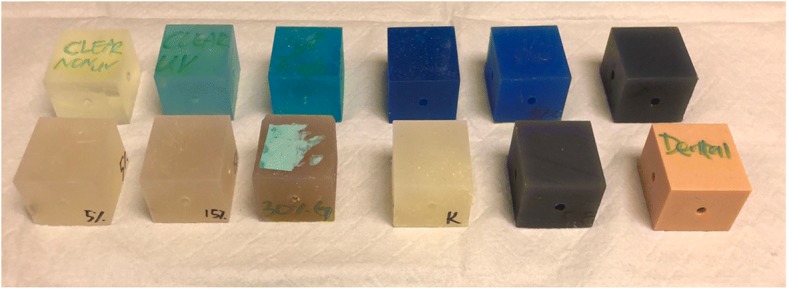
All 3DP cubes tested. Starting from the top left from left to right: clear non-UV, clear UV, 10% tough + 90% clear, 100% tough, 10% castable + 90% clear, 10% flexible + 90% clear, 5% gypsum + 100% clear, 15% gypsum + 100% clear, 30% gypsum + 100% clear, 0.125% Kevlar + 100% clear, 5% flexible + 5% tough + 90% clear, and 100% dental. Not pictured are the FDM-printed PLA and nylon. All blocks me*asured at 40 mm × 30 mm × 30 mm*

All samples unless otherwise stated were mixed with Clear® resin (i.e. 5% gypsum = 5% gypsum powder + 100% clear, 10% Castable® = 10% Castable + 90% Clear®). There was a total of 12 mixture combinations: 4 powder mixtures and 8 resin mixtures. The powder mixtures included 5% gypsum powder mix, 15% gypsum powder mix, 30% gypsum powder mix, and 0.125% Kevlar powder mix (see Table 1 and Fig. 6). The resin mixtures included 100% Clear® without UV-curing, 100% Clear® with UV-curing, 10% Tough®, 100% Tough®, 10% Castable®, 5% Flexible® + 5% Tough®, 10% Flexible®, and 100% Dental®. All samples were not UV-cured except for the first standard Clear® resin model; UV-curing at 405 nm is said to improve mechanical characteristics of the 3DP, increasing tensile failure, compression failure and bending failure significantly while increasing brittleness [11]. Therefore, we chose to UV-cure for only 10 min instead of the recommended 30 min since this also significantly increases the brittleness of the material, defeating the whole purpose of mimicking bone as closely as possible since bone is known to have anisotropic and dynamic properties with high tensile properties.

**Table 1:** All mixture combinations of both resin and powder types with their accompanying percentages. The resins used were provided from Formlabs® (Clear, Flexible, Castable, Tough, Dental) and powders were Gypsum and Kevlar. The last column shows whether or not the print was UV-cured. Percentage of powder was of total weight while percentage of resin was remaining

Resin 1	Resin 1%	Resin 2	Resin 2%	Powder Type	Powder %	UV (Y/N)
Clear	100	N/A	N/A	N/A	N/A	N
Clear	100	N/A	N/A	N/A	N/A	Y
Clear	90	Tough	10	N/A	N/A	N
Tough	100	N/A	N/A	N/A	N/A	N
Clear	100	N/A	N/A	Kevlar	0.125	N
Clear	90	Castable	10	N/A	N/A	N
Clear	100	/A	N/A	Gypsum	5	N
Clear	100	N/A	N/A	Gypsum	15	N
Clear	100	N/A	N/A	Gypsum	30	N
Clear	90	Flexible	10	N/A	N/A	N
Clear	90	Flexible/Tough	5 (Flex.), 5 (Tough)	N/A	N/A	N
Dental	100	N/A	N/A	N/A	N/A	N

Combinations involving powders Calcium Sulfate Gypsum and Kevlar (Learn to Brew LLC and DuPont USA®, respectively) were all SLA-printed from the Form 2® printer and mixed with the photopolymer resin solution prior to print, then poured into the resin tank for 3D-printing. The Gypsum and Kevlar are both powder forms that were weighed as a percentage of the total resin used, so any gypsum or Kevlar mixture includes 100% Clear® resin. Gypsum is a milled powder that generally has the responsibility of binding or solidifying components; this material was chosen due to the belief it would make the print harder without making it too brittle or catastrophically fail during printing. Kevlar is a common material most popularly known for its use in bullet proof vests, where this material was chosen for the same purposes as Gypsum— it would increase the hardness and mechanical properties of the print.

#### Structure: Compression combinations

**Fig. 7:**
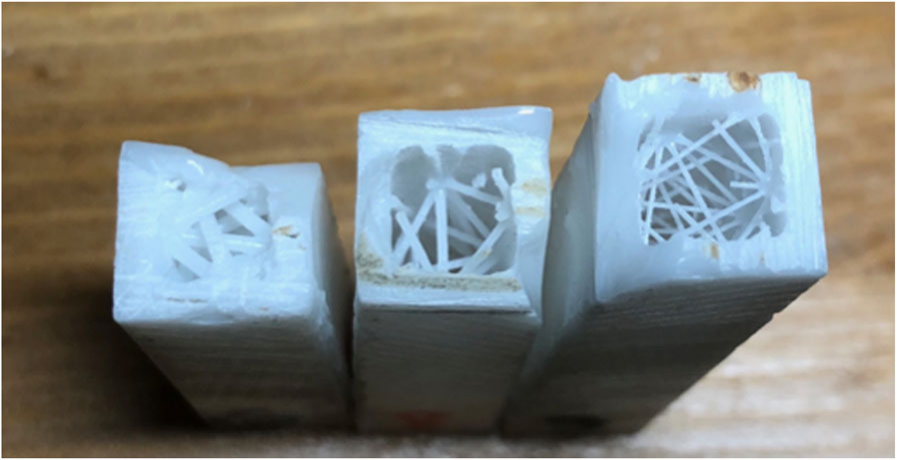
3 of 10 cubic blocks tested for structural mechanical characteristics. Differences in cylindrical diameters, cortical wall thickness, and gap sizes between cylinders c*an be seen between the 3 prints*

For this part of the model’s design, it is important to know there are essentially three changing parameters for the drill compression tests: cortical wall thickness, gap size of the cylinders in the internal structure, and radius of the internal cylinders. An algorithm using Python was written to add a random, stochastic framework of internal meshing within an empty cubic block using different combinations of ranges for the aforementioned parameters. The algorithm took a shell of an STL (stereolithography) file, which is commonly used for 3D-printing, and added these various combinations of the cylindrical pillars into the STL file in random positions and orientations every iteration (see Fig. 7). The algorithm was written to also allow the internal pillars to overlap, but not to the point of creating too thick of a solid since the goal was to the develop a random meshwork of pillars that are separated by incremental spaces.

For cortical wall thickness, the variations were 1.5 mm, 2 mm, 2.5 mm, and 3 mm thickness. For gap size, the different variations were 3 mm gap size, 4 mm gap size, 5 mm gap size, and 6 mm gap size. For radius of internal cylinders, 0.15 mm, 0.2 mm, 0.25 mm, and 0.3 mm radius were the changing variables. The exact combinations of 10 separate prints can be seen in Table 2. Since SLA printing begins with a liquid resin, escape holes were implemented into the designs of the vertebrae models in order to allow any access liquid resin to leak out. Models were also put into a vacuum-sealed chamber for 30 min after post-curing, allowing both the external and internal structure of the model to fully dry. The cylinders were completely solid which means no escape holes were needed for the cylinders themselves.

**Table 2:** This chart displays the maximum peaks of each material combination in force in the x-direction during the bur sweep testing. Force/maximum peak is in Newtons

Force in X-Direction (Fx)		
Material Combination	Maximum Peak (N)	vs. Vertebrae (N)
Non-UV	44.5	11.9
UV	46.1	13.5
10% Tough + 90% Clear	10.8	−21.8
100% Tough	45.6	13
0.125% Kevlar + 100% Clear	44.8	12.2
10% Castable + 90% Clear	27.6	−5
5% Gypsum + 100% Clear	40.2	7.6
15% Gypsum + 100% Clear	27.8	−4.8
30% Gypsum + 100% Clear	20.9	−11.7
10% Flexible + 90% Clear	44.3	11.7
5% Flexible + 5% Tough + 90% Clear	21.4	− 11.2
100% Dental	46.5	13.9
PLA Unpolished	21	−11.6
PLA Polished	19.8	−12.8
Nylon	50.4	17.8
Vertebrae	32.6	0

These values and parameters were chosen after several back and forth meetings with the neurosurgeons from AGH working closely on the project in this study. Initial frameworks included different parameters and measurements. The shape of pillars was chosen since we believed this best mimicked the shape of trabecular, spongy bone that the printer could successfully print due to its round and curved geometry. Further changes and tinkering of the parameters were done after surgeons gave qualitative feedback on how the trabecular 3D-print felt compared to real, human bone. After several meetings and attempts of developing accurate parameters, a range was chosen to give the surgeons more options in terms of what may have felt similar to human bone while achieving a geometry that could be both similar to what is demonstrated in trabecular bone and printable without failure due to various reasons such as the cylinders being too small, spaced apart, or not enough supports.

### Cadaveric specimens

**Fig. 8:**
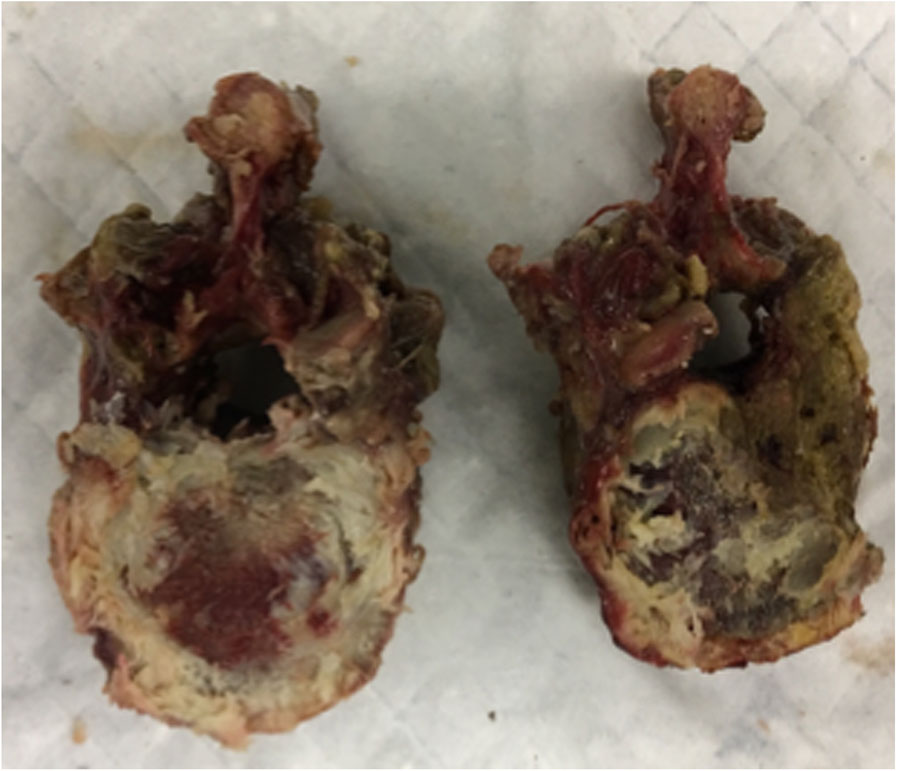
Intervertebral discs on the human cadaveric vertebrae were shaved down using cuvettes and scalpels in order to expose as much bone (endplate) as possible to bett*er compare to the 3DP specimens*

A total of 10 human, cadaveric thoracic vertebrae were individually segmented. 8 were used for the bur sweep test and 2 were used for the drill compression test. The specimens underwent one freeze-thaw cycle in which they were taken out to thaw, then cut and cleaned down to the endplates, put in the fridge to maintain biomechanical characteristics, then subsequently tested the next day. They were from two different male human donors, both over 50 years of age. In order to better compare properties of bone, the intervertebral discs which is cartilaginous tissue lying between vertebrae, were sheared and cut until bone was visible using a cuvette, a medical instrument primarily used to shave down bone or tissue (see Fig. 8).

### Meshing Algorithm & Data Analysis

Data analysis for the bur sweep data was done using Microsoft Excel® and MATLAB®. The original data was converted (voltage to Newtons) and tabulated into Excel® while a script was written MATLAB® to take this raw data and develop the accompanying graphs that can be seen in the results. The eight vertebrae used for the bur sweep were tested individually then averaged. Lastly, each material combination was tested only once. Several two sample t-tests were performed to show if statistical significance existed between the material combinations and the vertebrae, where the null hypothesis was they were equal and the alternate hypothesis was they were not equal. All *p*-values for each comparison of a sample to vertebrae were less than 0.05, meaning the null hypothesis that they are equal equal was rejected.

The internal mesh algorithm was written using Python. An empty shell or object in an STL file is needed prior to running this algorithm. The STL shell is taken and the algorithm fills the object with an external wall then a random, stochastic network of pillars and cylinders with varying parameters such as radius of cylinders, gap sizes between cylinders, and a wall surrounding these cylinders. The parameter values can be changed by the user within the code and the cylinders are allowed to overlap but not more than once or else it would create a solid clump, defeating the whole purpose of developing a mesh. Each particular combination for drill compression testing as seen in Table 3 was tested three times each (always drilling in the midpoint of the cubic block) then averaged. The two vertebrae for drill compression were tested then averaged.

**Table 3:** All printed combinations of varying internal and external structure parameters for drill compression test. Each row represents a single print, totaling 10 different prints with varying parameters. All cubic blocks were printed at 0.1 mm resolution on Form 2 Printer®

Drill Compression Combos		
Cortical Wall Thickness (mm)	Gap Size (mm)	Radius of Cylinder (mm)
1.5	4	0.2
2	3	0.2
2	4	0.15
2	4	0.2
2	4	0.25
2	4	0.3
2	5	0.2
2	6	0.2
2.5	4	0.2
3	4	0.2

## Results

### Material: Bur sweep force results

**Fig. 9:**
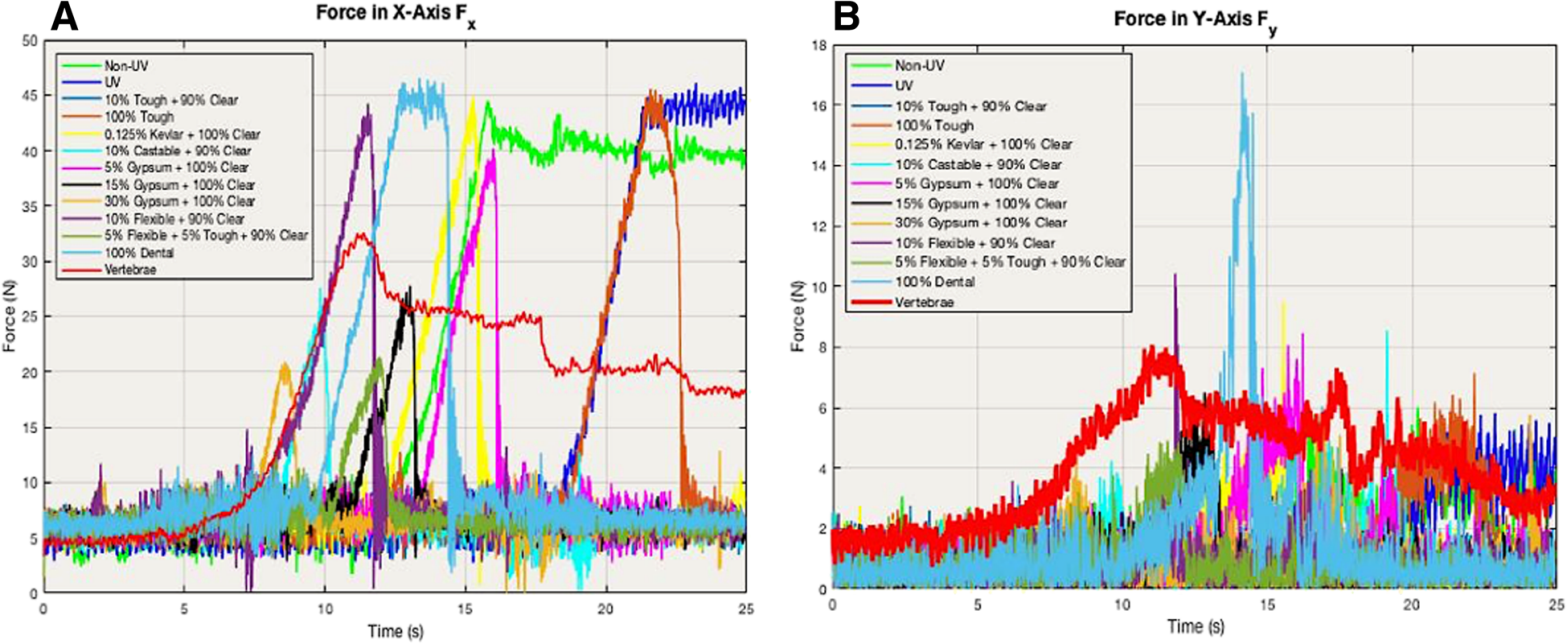
(**a**) Force in x-axis direction Fx and (**b**) force in y-axis direction Fy. The x-axes in the graphs depict time (s) and y-axes is force in Newtons. It can be assumed if the rest of the percentages are clear if it is a resin (i.e. 10% castable and 90% clear) and 100% clear if it is a powder (gypsum, Kevlar). The spectra are not completely overlapped due to minuscule varying placements of the specimens during testing in the sagittal and coronal planes. A few millimeters difference in position can lead to a difference in when the bur hits the sample

**Fig. 10:**
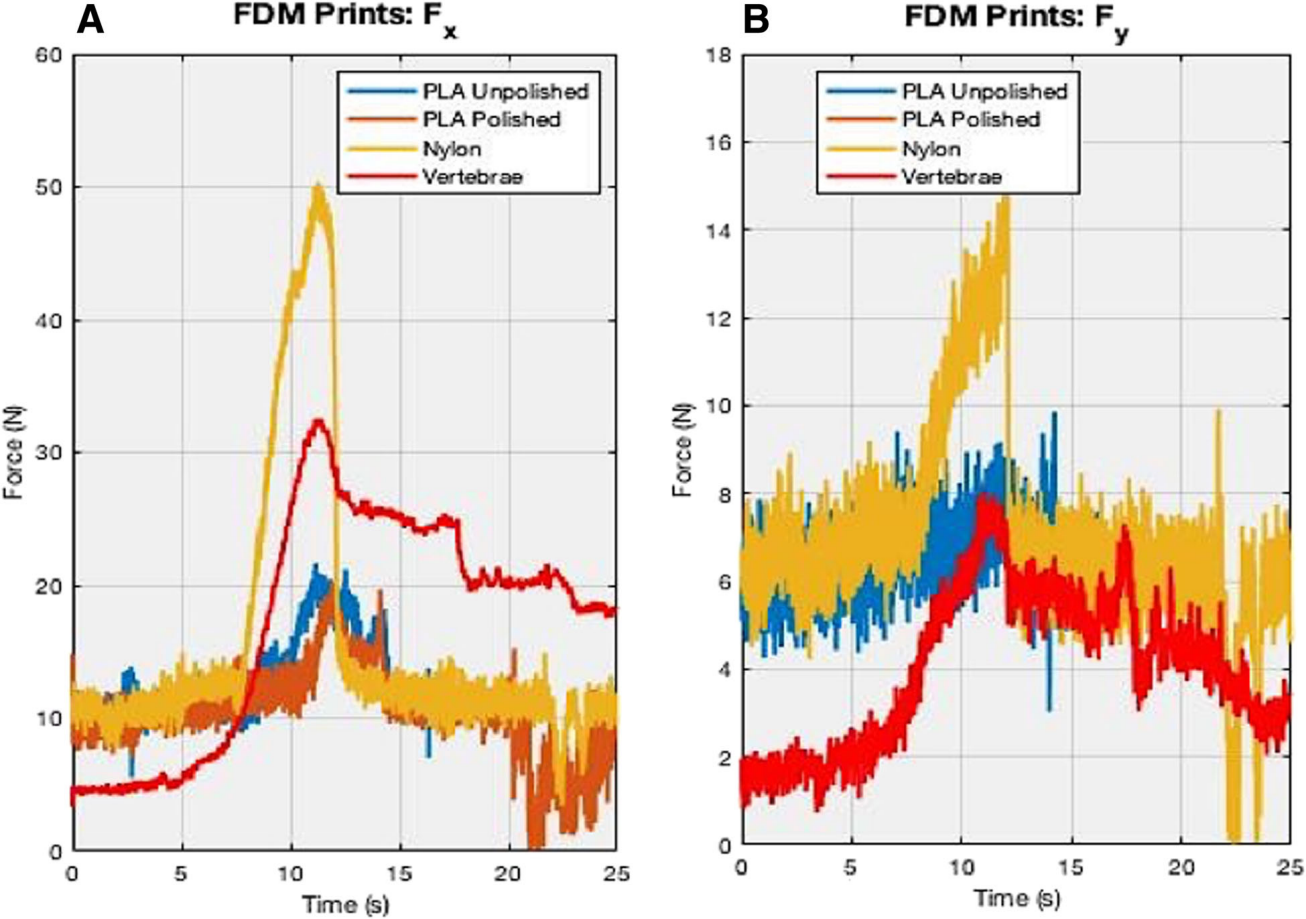
These graphs show the comparisons of the FDM-printed PLA unpolished, PLA polished, and nylon to that of the human cadaveric vertebrae for force in both the (**a**) x and (**b**) y-directions. The x-axis is time (in seconds) and y-axis is Force (in Newtons). The spectra are not completely overlapped due to miniscule varying placements of the specimens and time lag during testing. A few millimeters difference in position can lead to a difference *in when the bur hits the sample*

If the rest of the mixture’s type and percentage is not otherwise stated, it can be assumed it is clear (i.e. 10% Castable® is 10% Castable® mixed with 90% Clear®). Powders were measured and mixed by weight, so 15% gypsum powder means 15% gypsum powder mixed with 100% Clear® resin. Furthermore, forces are sensed at different times due to the slight differences in positioning of each sample. Although every sample’s transverse plane was parallel with the floor, same exact positioning in the saggital and coronal plane of every single sample was unattainable. The values of the forces, specifically the peaks that can be seen in Figs. 9 and 10, are the maximum forces the bur encountered while sweeping across the sample. Again, the x-direction represents the direction the bur is sweeping as it goes across the vertebrae or printed piece (x-axis parallel to direction of bur sweep), whereas the y-direction is representative of the specimen’s axis that is perpendicular to the x-direction which is why both the forces and moments in the y-directions are lower and have a lower signal-to-noise ratio.

**Fig. 11:**
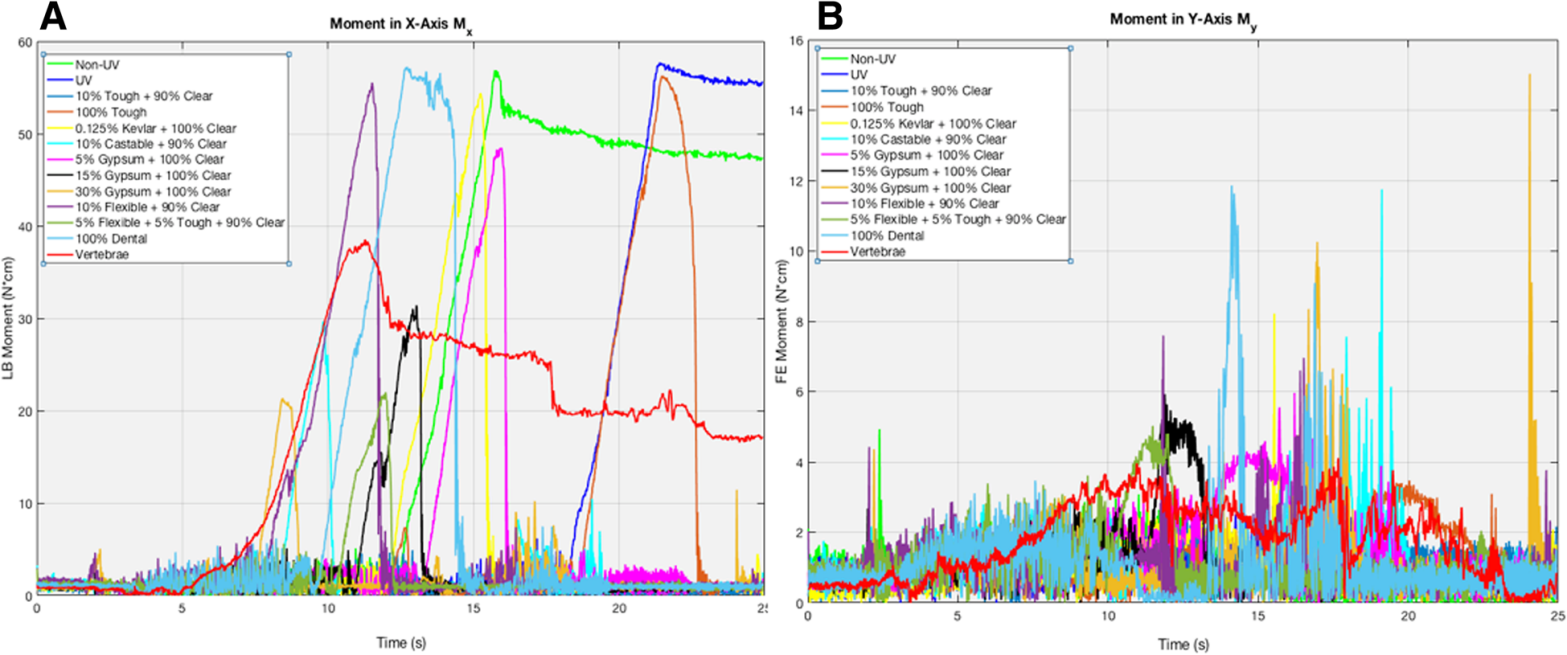
(**a**) Moment in x-axis direction Fx and (**b**) Moment in y-axis direction Fy. The x-axes in the graphs depict time (s) and y-axes is force in N-cm. It can be assumed if the rest of the percentages are clear if it is a resin (i.e. 10% castable and 90% clear) and 100% clear if it is a powder (gypsum, Kevlar). The spectra are not completely overlapped due to minuscule varying placements of the specimens during testing in the sagittal and coronal planes. A few millimeters difference in position can lead to a difference *in when the bur hits the sample*

The reaction forces shown in the results demonstrate that 15% gypsum powder (black in Fig. 9) and 10% Castable® (light cyan in Fig. 9) were closest to the human cadaveric vertebrae (red in Figs. 9 and 11), especially force in the x-direction, F_x_ where 15% Gypsum had a maximum value at 27.8 N, 10% Castable had a maximum value of 27.6 N with vertebrae’s maximum value at 32.6 N (see Fig. 9a and Table 2). Non-UV Clear®, UV Clear®, 100% Tough®, 0.125% Kevlar powder and 10% Flexible all reached a maximum of approximately 44–46 N. 5% gypsum powder was higher than 15% and 30% gypsum powder, reaching a maximum of 40.2 N. The 5% Flexible® + 5% Tough® mixture and 30% gypsum powder combinations were the lowest, reaching a maximum of only 20 N (see Fig. 9a and Table 2)*.* FDM comparisons showed nylon was in fact higher by approximately 30 N than both PLA prints, reaching a maximum value of 50.4 N for in F_x_ (see Fig. 10a and Table 2). There were little to no differences between the PLA unpolished and polished prints in comparison to each other, both peaking at approximately 20 N and both being less than the 32 N cadaveric vertebrae. Two sample t-tests showed statistical significance, thus rejecting the null hypothesis that any of these combinations were equal to the vertebrae (*p* < 0.05) (see Table 4).

**Table 4:** All tabulated results of p-values calculated from a two sample t-test to compare significance between each of the material combinations to the vertebrae for each of the bur sweep’s parameters (F_x_, F_y_, M_x_, and M_y_)

*p*-values (Two-Sample Test)	Fx	Fy	Mx	My
Non-UV	4.00E-18	0.00E + 00	1.81E-27	9.64E-241
UV	3.73E-34	0.00E + 00	2.14E-20	1.66E-233
10% Tough + 90% Clear	0	0	0	3.31E-178
100% Tough	2.79E-148	0.00E + 00	2.37E-151	3.23E-139
0.125% Kevlar + 100% Clear	4.14E-240	0.00E + 00	3.06E-252	3.22E-277
10% Castable + 90% Clear	0	0	0	2.18E-114
5% Gypsum + 100% Clear	7.00E-285	0.00E + 00	1.26E-312	3.24E-30
15% Gypsum + 100% Clear	0	0.00E + 00	0	4.21E-91
30% Gypsum + 100% Clear	0	0	0	5.55E-72
10% Flexible + 90% Clear	2.43E-217	0.00E + 00	1.00E-231	4.68E-159
5% Flexible + 5% Tough + 90% Clear	0	0.00E + 00	0	3.97E-84
100% Dental	7.50E-75	0	8.00E-92	6.91E-46
PLA Unpolished	7.75E-85	0.00E + 00	0	8.44E-94
PLA Polished	3.67E-256	0.00E + 00	0	2.48E-05
Nylon	6.87E-10	0	8.77E-130	1.49E-17

However, the force in the y-direction demonstrated noisy results (F_y_). Again, this was expected since the majority of movement is the bur sweeping in the x-coordinate and x-direction, which means there would only be small amounts of force in the y-direction, as is shown in Fig. 9b. The free-body diagram in Fig. 4 also demonstrates the movement of the bur going in the x-direction. Vertebrae reached a maximum of 8.1 N, while 10% Flexible® + 90% Clear® and 100% Dental® reached maximums of 10.4 N and 17.1 N, respectively (see Fig. 9b and Table 5). All other combinations stayed within 5–9 N. Two sample t-tests showed statistical significance, thus rejecting the null hypothesis that any of these combinations were equal to the vertebrae (p < 0.05) (see Table 4).

**Table 5:** This chart displays the maximum peaks of each material combination of force in the y-direction during the bur sweep testing. Force/maximum peak is in Newtons

Force in Y-Direction (Fy)		
Material Combination	Maximum Peak (N)	vs. Vertebrae (N)
Non-UV	6	−2.1
UV	5.8	−2.3
10% Tough + 90% Clear	2.8	−5.3
100% Tough	7.1	−1
0.125% Kevlar + 100% Clear	9.5	1.4
10% Castable + 90% Clear	8.6	0.5
5% Gypsum + 100% Clear	8.5	0.4
15% Gypsum + 100% Clear	6.5	−1.6
30% Gypsum + 100% Clear	5.8	−2.3
10% Flexible + 90% Clear	10.4	2.3
5% Flexible + 5% Tough + 90% Clear	4.9	−3.2
100% Dental	17.1	9
PLA Unpolished	9.2	1.1
PLA Polished	8	−0.1
Nylon	15.5	7.4
Vertebrae	8.1	0

### Material: Bur sweep moment results

**Fig. 12:**
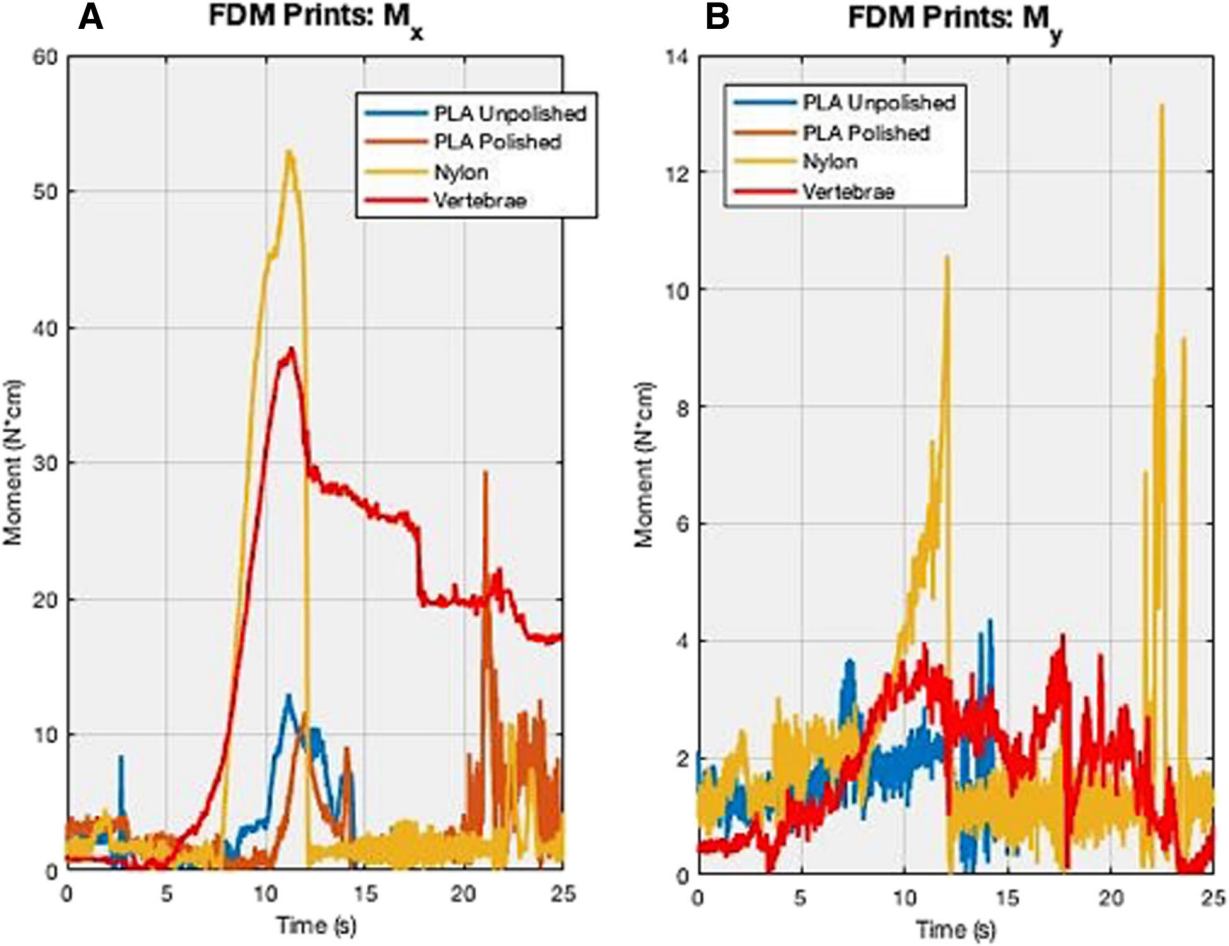
These graphs show the comparisons of the FDM-printed PLA unpolished, PLA polished, and nylon to that of the human cadaveric vertebrae for moment in both the (**a**) x and (**b**) y-directions. The x-axis is time (in seconds) and y-axis is Moment (in Newton-cm). The spectra are not completely overlapped due to miniscule varying placements of the specimens and time lag during testing. A few millimeters difference in position can lead to a difference *in when the bur hits the sample*

The moments’ data is not perfectly aligned due to being sensed at different times from the slight differences in positioning of each sample. Although every sample’s transverse plane (top surface) was parallel with the floor, same exact positioning in the saggital and coronal planes of every single sample was unattainable due to slight position adjusting, calibration requiring moving the sample, etc.. The values of the moments (N-cm), specifically the peaks that can be seen in Figs. 11 and 12, is the maximum amount of force that can be attained while attempting to rotate a specific point or axis, where this point changes continuously as the bur sweeps.

Based on the lateral bending moment (x-direction) results in Fig. 11a above, 15% gypsum powder (black in Fig. 11) and 10% Castable® (light cyan in Fig. 11) were again closest to the vertebrae (red in Fig. 11) in the lateral bending moment, M_x_, with a difference of 7.1 N-cm and 8.7 N-cm compared to the vertebrae’s maximum M_x_, respectively (see Table 6). Vertebrae had a maximum value at 38.5 N-cm, while 15% gypsum powder and 10% Castable® had maximum values at 31.4 N-cm and 29.8 N-cm, respectively. The results were furthermore analogous to the F_x_ results in regard to following a similar trend, where 10% Flexible®, 100% Dental®, non-UV Clear®, UV-Clear®, 0.125% Kevlar powder, and 100% Tough® were all higher than vertebrae by a magnitude of about 18 N-cm, where the aforementioned combinations had maximum values in a range of 54–56 N-cm (see Fig. 11a and Table 6). Once more, 30% gypsum powder and 5% Flexible® + 5% Tough® were lower than vertebrae by a magnitude of approximately 16 N-cm, both only peaking at 21 N-cm. 5% gypsum powder’s peak value was again in between vertebrae and the highest of the aforementioned combinations, reaching a maximum of approximately 48 N-cm, showing a similar pattern to the F_x_ results (see Fig. 11a and Table 6). The FDM printed results showed that nylon reached a maximum of approximately 53 N-cm, PLA unpolished reached a maximum of 12.2 N-cm, and PLA polished reached a maximum of 29.7 N-cm (see Fig. 12a). Two Sample t-tests showed statistical significance, thus rejecting the null hypothesis that any of these combinations were equal to the vertebrae (*p* < 0.05) (see Table 4).

**Table 6:** This chart displays the maximum peaks of each material combination of moment in the x-direction during the bur sweep testing. Moment/maximum peak is in Newton-centimeters

Moment in X-Direction (Mx)		
Material Combination	Maximum Peak (N-cm)	vs. Vertebrae (N)
Non-UV	56.9	18.4
UV	57.7	19.2
10% Tough + 90% Clear	4.9	−33.6
100% Tough	56.2	17.7
0.125% Kevlar + 100% Clear	54.5	16
10% Castable + 90% Clear	29.8	−8.7
5% Gypsum + 100% Clear	48.5	10
15% Gypsum + 100% Clear	31.4	−7.1
30% Gypsum + 100% Clear	21.3	−17.2
10% Flexible + 90% Clear	55.5	17
5% Flexible + 5% Tough + 90% Clear	22	− 16.5
100% Dental	57.2	18.7
PLA Unpolished	12.2	− 26.3
PLA Polished	29.7	−8.8
Nylon	52.7	14.2
Vertebrae	38.5	0

The M_y_ results were expected to show significant noise, similar to the F_y_ results but still has a higher signal-to-noise ratio than the F_y_ results. Despite this, it was expected M_y_’s maximum values would be generally low in respect to values in the M_x_ direction, as high of a difference as 55 N-cm. 10% Castable® and 100% Dental® reached a maximum of 11.9 N-cm while 30% gypsum powder reached a maximum of 15 N-cm (see Fig. 11b and Table 7). Vertebrae reached a maximum then plateaued around 4 N-cm. 10% flexible reached a maximum of 7.6 N-cm and 15% gypsum powder reached a maximum of 6 N-cm (see Fig. 11b). 5% gypsum, 5% Flexible® + 5% Tough®, 10% Flexible®, and Clear® non-UV were closest to vertebrae’s peaks, all reaching maximum values at 5–6 N-cm (see Fig. 11b and Table 7). For FDM prints, nylon reached a maximum of 13.4 N-cm for M_y_, while PLA unpolished and polished both reached maximum values at 4.2 N-cm (see Fig. 12b and Table 7). Two sample t-tests showed statistical significance, thus rejecting the null hypothesis that any of these combinations were equal to the vertebrae (p < 0.05) (see Table 4).

**Table 7:** This chart displays the maximum peaks of each material combination of moment in the y-direction during the bur sweep testing. Moment/maximum peak is in Newton-centimeters

Moment in Y-Direction (My)		
Material Combination	Maximum Peak (N-cm)	vs. Vertebrae (N)
Non-UV	4.9	0.8
UV	3.2	−0.9
10% Tough + 90% Clear	3	−1.1
100% Tough	3.5	− 0.6
0.125% Kevlar + 100% Clear	8.2	4.1
10% Castable + 90% Clear	11.8	7.7
5% Gypsum + 100% Clear	6	1.9
15% Gypsum + 100% Clear	6	1.9
30% Gypsum + 100% Clear	15	10.9
10% Flexible + 90% Clear	7.6	3.5
5% Flexible + 5% Tough + 90% Clear	5	0.9
100% Dental	11.9	7.8
PLA Unpolished	4.2	0.1
PLA Polished	4.2	0.1
Nylon	13.4	9.3
Vertebrae	4.1	0

### Structure: Compression test results

**Fig. 13:**
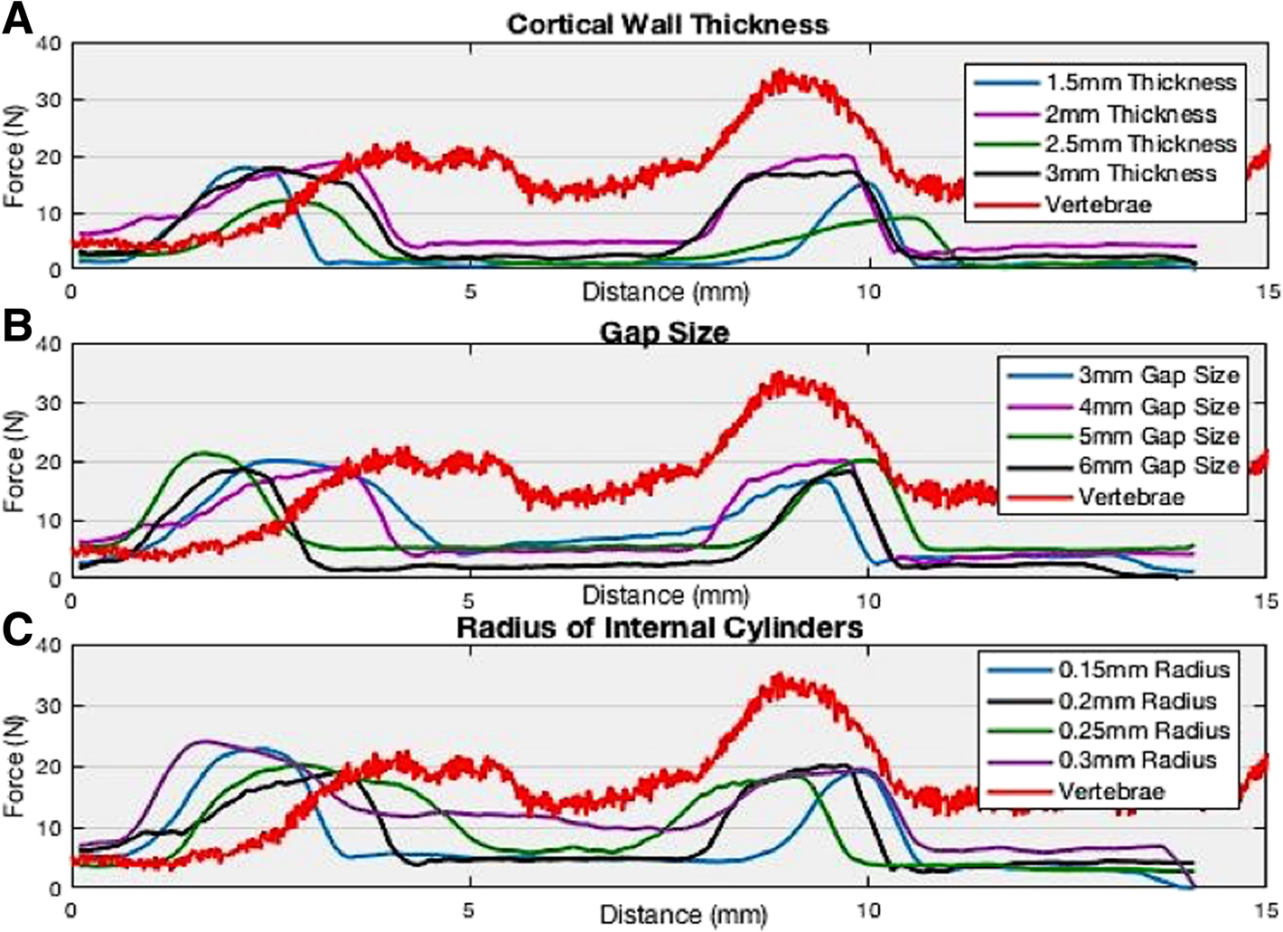
Results of internal structure force compression. They are broken up top to bottom as (**a**) cortical wall thickness, (**b**) gap size, and (**c**) radius of internal cylinders, where each chart varies with dimen*sions of the targeted parameter*

The compression test results are grouped into three different groups: cortical wall thickness, gap size between cylinders, and radius of the cylinders. It can be assumed for each of the graphs, the first peak is when the drill pierces the first outer wall, followed by going through internal structure, then going through the second outer wall, completely drilling through the print (see Fig. 13). The vertebrae’s results demonstrated a peak 1 value of 20.9 N, an amplitude of 4.9 mm and a peak 2 value of 33.8 N.

Cortical wall thickness shows that 2 mm is closest to vertebrae with a peak 1 value of 18.4 N and peak 2 value of 20.2 N (see Fig. 13a and Table 8). Gap size results demonstrate that 5 mm was the closest to vertebrae with a first peak value of 20.8 N, amplitude of 8.5 mm, and a second peak value of 21.3 N (see Fig. 13b and Table 8). However, 4 mm gap size does show the lowest amplitude of 6.3 mm closest to the vertebrae’s 4.9 mm. Lastly, radius of internal cylinders demonstrates that although 0.25 mm or 0.3 mm radius of cylinders are closest to the vertebrae with 0.25 mm radius of cylinder’s first peak at 18.3 N, amplitude of 6.2 mm, and second peak of 20.2 N. The first peak of 0.3 mm was 19.3 N, amplitude of 8.4 mm, and second peak of 23.4 N (see Fig. 13c and Table 8). Based on the combinations available and tested, the best fit for cortical wall thickness, gap size, and radius of cylinders from the given and tested combinations are 2 mm thickness, 4 or 5 mm gap sizes, and 0.25 mm radius of internal cylinders.

**Table 8:** Results of cortical wall thickness, gap size, and radius of cylinders’ two peaks and amplitudes for each corresponding size. Peak 1 is the first peak in Fig. 13 and peak 2 is the second peak in Fig. 13, both measured in Newtons. Amplitude is measured in millimeters from peak-to-peak. Vertebrae can be seen in the last row of Table 8

Type	Size (mm)	Peak 1 (N)	Amp (mm)	Peak 2 (N)
Cortical Wall Thickness	1.5	17.9	7.7	15.3
Cortical Wall Thickness	2	18.4	6.4	20.2
Cortical Wall Thickness	2.5	11.9	6.3	10.5
Cortical Wall Thickness	3	17.8	7.1	17.2
Gap Size	3	20	7.3	16.6
Gap Size	4	18.9	6.3	20.2
Gap Size	5	20.8	8.5	21.3
Gap Size	6	18.6	7.6	19.9
Radius of Cylinder	0.15	19.4	7.5	22.9
Radius of Cylinder	0.2	19.2	6.4	18.9
Radius of Cylinder	0.25	18.3	6.2	20.2
Radius of Cylinder	0.3	19.3	8.4	23.4
Vertebrae	–	20.9	4.9	33.8

## Discussion

It was hypothesized and expected that force and moment in the y-direction would yield a low signal-to-noise ratio as evidenced in the free-body-diagram. The type motion being investigated is a sweeping arc of the drilling bur which means one plane, in this case the transverse or the x-direction plane, will yield the more pronounced results with larger values for both force and moments as well as an improved signal-to-noise ratio since there is little to no movement in the y-direction if the sweep is going in only the x-direction.

The force and moment in the x-direction show very similar trends, where 15% gypsum + 100% Clear and 10% Castable and 90% Clear are closest to vertebrae for both the force and moment in the x-direction (see Figs. 9a and 11a). This is expected since force and moment will be expected to increase in the plane that the motion is being observed in. For both force and moment in the x-direction, 15% gypsum (black in Figs. 9 and 11) and 10% castable (light cyan in Figs. 9 and 11) are both closest to vertebrae (red in Figs. 9 and 11). The reason the non-uv (green in Figs. 9 and 11) and UV (blue in Figs. 9 and 11) specimens plateau whereas the others do not is because the positioning of these two particular specimens were slightly higher than the rest, which means the bur had more space on the specimen to sweep across, leading to a plateau as the bur simultaneously continues drilling and digging through the specimen. However, the peaks of each of the samples in the graphs is of utmost prevalence since it signifies maximum force or moment the specimen undergoes when a bur is being applied in an arc-like motion similar to surgeons’ practice. The slowly decreasing plateau of the vertebrae (red in Figs. 9, 10 and 11) is attributed to the imperfections readily apparent in human anatomy, where even after the intervertebral disc is shaved down to the endplates, there still exists bumps and unevenness of bone, leading to not just one peak, but potentially multiple peaks, whereas the 3D-printed specimens could be printed flat.

Interestingly, it seems that the non-UV cured print is far too high compared to the vertebrae by a magnitude of 11.9 N in F_x_ and 18.4 N-cm in M_x_, along with several other combinations of materials such as 100% Dental, UV cured clear, 10% Flexible + 90% Clear, and 100% Tough (see Figs. 9a and 11a and Tables 2 and 4). The prints including Tough® were expected to be higher than vertebrae since the Tough® material is made for durable and stronger required materials such as jet engine components with a flexural modulus of 0.6 GPa [11]. However, 10% flexible combination prints were unexpected since Flexible® material is said to have a higher elongation to failure value at 60%, thus the assumption the resistance to the bur drilling would be lower. Formlabs™ claims that UV-curing significantly improves mechanical characteristics, but in turn does decrease elongation at failure, making it more brittle which could explain why that non-UV is still above vertebrae and below the UV-cured print [11].

Furthermore, 30% gypsum was lowest out of the powder mixes at 20.9 N in F_x_ and 21.3 N-cm in M_x_. This was unexpected since it was hypothesized that including more gypsum would in fact harden the material (see Tables 2 and 4). This could be due to the gypsum powder hitting a certain threshold before the mixing and bonding of the gypsum while clear photopolymer resin yields a larger breakdown of the material, making the material less tough. This does tie in with the other values since 5% gypsum is shown to be the highest, while 15% lies in the middle, showing a direct trend of increasing gypsum powder yielding lower resistance to bur sweeping motion. From this, it can be inferred that somewhere between 15 and 30% gypsum with 100% clear resin mixture yields the optimal threshold in terms of not being too brittle while showing high enough resistance to drilling.

It is a positive sign that both the lateral bending moment M_x_ and force in the x-direction F_x_ have nearly identical trends in terms of where the peaks of the printed combinations lie in respect to the vertebrae’s peak. Based on these results, it seems that both 15% gypsum with 100% Clear® and 10% Castable® mixed with 90% Clear® are the best and optimal mixtures when it comes to mimicking vertebral bone for this particular bur-sweeping motion. It can be furthermore argued that finding a gypsum mixture somewhere between 5 and 15%, perhaps 8–10% could yield an even more accurate overlapped spectra of material properties with that of bone since the 5% gypsum mixture’s values are above the vertebrae’s forces and moments while the 15% gypsum mixture is below.

Moreover, the FDM print comparisons shows that nylon is significantly higher than the vertebrae, for both F_x_ and M_x_, lying in the same range values as the other higher mixtures such as 10% tough, 10% flexible, etc. F_x_ for nylon, PLA unpolished, PLA polished had maximum values at 50.4 N, 21 N, and 19.8 N, respectively while the M_x_ had maximum values at around 52.7 N-cm, 12.2 N-cm, and 29.7 N-cm, respectively (see Figs. 10 and 12 and Tables 2 and 4). This illustrates PLA polished and unpolished have little to no differences while PLA and nylon both are not good fits with that of human bone when it comes to the bur sweeping motion.

In regard to the drill compression results, although the results do not exactly match up with that of the vertebrae, the trends prove similar, as well as the magnitudes of the changing parameters not being far off. For cortical wall thickness, it can be argued that 2 mm thickness is the best candidate for a best fit due to holding the highest peak and largest amplitude compared to the vertebrae (see Fig. 13a). Gap size radius demonstrates that 4 mm and 5 mm are the best candidates due to their large amplitudes, where 4 mm gap size shows a slight increase in the trabecular region (middle of the peaks), meaning that the load cell is in fact sensing an increase in force but still lower than the peaks of the cortical walls (see Fig. 13b). Lastly, radius of internal cylinders show that 0.25 mm and 0.3 mm radii are closest to the vertebrae, both of which have the largest amplitudes at the cortical walls while 0.3 mm has the highest force in the middle between the two peaks, coming closest to the vertebrae, off by only 2–4 N (see Fig. 13c). Therefore, the best combination based on these results for cortical wall thickness, gap size, and radius of internal cylinders are 2 mm thickness, 3–4 mm gap size, and 0.25–0.3 mm radius of cylinders. However, there is work left to be done in terms of finding a better fit since there are several more values and parameters that can be changed and the peaks and amplitudes do not fully align with that of the human vertebrae’s force compression values.

Despite the promising results, there still remains work to be done to find the best material and internal structure combination. In terms of material, finding a gypsum combination between 5 and 15% mixed with clear resin or varying combinations of castable with perhaps gypsum need to be further investigated. Internal structure parameters need to be changed as well where the prints could be perhaps printed at higher resolution so cortical wall thickness from 2 to 2.5 mm thickness can be investigated, gap sizes of 3–4 mm, and radius of internal cylinders between 0.25–0.3 mm.

Further work will involve a qualitative study with participants including several residents, clinicians, and practicing neurosurgeons. They will be given the samples similar to this mechanical study and will rate them on a given scale from 1 to 5 for categories of similar feel to human bone using the bur, the cortical to cancellous transition using the bur, instrumentation using a kerrison to break off the print, and visual appearance. This should shed light on any internal consistency that will exist between the results in this study and the qualitative study in the future. The material’s bur sweep results can be argued to demonstrate that a method has been developed to quantify tactile fidelity as well as a drill compression test to validate similarities between trabecular and cortical shells of real, human bone to that of a 3D-printed specimen.

## Limitations

Unfortunately, the bur sweep test is a complex motion being investigated, requiring an extremely responsive DAQ for the several values of moments and forces being measured and quantified simultaneously. This in turn could lead to lots of noise, as seen in the figures above. Smoothing was done in MATLAB to address this but could only aid in reducing noise to a certain extent. Using a more sensitive and lower load cell, as opposed to the 10 kN load cell could yield a higher sensitivity when investigating lower loads such as this. Furthermore, the bur sped at an extremely high rate (75,000 RPM). This rate was again chosen since it is roughly around the speed orthopedic and neurosurgeons use to drill through bone during surgery. However, when wanting to measure forces, moments, and other variables to high accuracy, adding a high-speed torque and movement has potential to increase the noise significantly, aiding to a blurrier picture of data.

The load cell principle also applies to the drill compression test where a 10 kN load cell can argued to be too high since the forces being investigated lie in the range of 0–30 N. Another limitation for the drill compression test is there are several more combinations and variations that could have been tested; we can observe the small forces that occur between the cortical shells, but ultimately, we want that to increase as much as possible while not surpassing the forces seen when the drill goes through the cortical wall (the two highest peaks in the graphs). There also comes the potential problem of stability, where since we are incorporating a rotating movement, it adds potential for more noise or less accurate results; it could have been possible the rotations could have yielded higher noise.

Another limitation is there was only one size and type of drill-bit used, since this is the predominant size and type used in most operating rooms (ORs) by surgeons attending AGH (5 mm diamond-coated bur tip). With this, only one speed RPM of 75,000 was investigated for the bur sweep since this is once more roughly the speed surgeons use in the OR. Future studies can investigate the impact of different drill bit sizes and RPM speeds when it comes to bur sweeping for 3DP and vertebrae. Furthermore, apart from experimenting with varying bur tips or RPM speeds, changes in the rate of the sweeping arc and its resultant forces and moments could also be investigated in the future. Since we only investigated 1.5 mm/s feed rate of the sweep, varying feed rates with both the bur sweep and the compression could potentially yield different results.

Moreover, prior to choosing the appropriate parameters for the internal structure of cylinders and the accompanying cortical wall thicknesses, surgeons and researchers had several meetings and discussions in order to map out the best fit combinations. There was no quantitative method involved in choosing the combinations, but simply qualitative feedback from surgeons that led to narrowing down of the internal structure parameters. The information that was important in these meetings was how close were the prints to real, human bone and how visually accurate were they? This included the cortical wall thickness, the appearance of the trabecular meshing, how the 3D-printed structures felt upon burring at 75,000 RPM and how well they could be instrumented such as using a kerrison or pliers to break off pieces. This is a limitation because it can be argued as subjectivity which led to these parameters, not necessarily a quantitative method to ascertain these combination sizes for the structure of the 3D-printed specimens.

Lastly, although the 3DP model we developed aimed to mimic a cortical to cancellous/trabecular bone structure, these models were only tested against human vertebrae. Although it can be argued vertebrae does in fact mimic bone due to the hard endplate surfaces and the anisotropic structure housed within, this certainly does not imply that the vertebrae’s bone will have the same exact hardness, resistance to the bur, or mechanical integrity as other bones in the body. It is possible that if the 3DP models were compared instead to bone in other areas of the body, the results may vary. Cadaveric human vertebrae were used because it was the most readily available, cheapest, and granted access via the hospital.

## Conclusion

Based on the results, the most appropriate material combinations given our attempts was that of 15% gypsum mixed with 100% clear and 10% castable mixed with 90% clear. The best internal structure fit was a 2 mm cortical wall thickness, 3 mm gap size, and 0.3 mm radius of internal cylinders. There remains more work to be done on finding a closer material combination, but it lies between the two mixtures of 15% gypsum and 10% castable, while improving resolution of internal structure could yield closer results to matching the mesh structure of human bone.

However, this study has allowed to paint a clearer picture in terms of what types of SLA and FDM printing combinations and materials work and do not work, along with the beginnings of exploration in both internal structure and 3DP to match that of human bone since neither has been studied extensively in literature. Comparing the mechanical results here to that of the future qualitative study involving surgeons and residents rating the similar 3DP materials will paint a clearer picture in regard to whether or not the qualitative study of the clinicians’ tactile feel using a bur themselves will reflect the mechanical results in this paper.

## Appendix

This section shows any extra figures or image that were not otherwise explicitly specified within the protocol. The 3DP vertebrae shapes that were also printed are shown here, with a transverse cut in one of the vertebrae showing an internal mesh trabecular structure. The type of bur and its motor can also be seen, which is similar to the instruments used in the OR (operating room).

Further specifications such as the shape of the cylindrical blocks used for the 1000 RPM compression testing is also shown, with accompanying dimensions using a standard caliper measured in mm. A closer image of the cube used for the bur sweep test can also be seen where each face of the cube has a small indented hole at the same spot which is where the all-thread screws would clamp, meaning every position of the clamped cube was the exact same on every test, reducing any potential for position or human error.

## Abbreviations

3DP: 3D-printed
AGH: Allegheny General Hospital
cm: centimeters
CMU: Carnegie Mellon University
FDM: fused deposition modeling
F_x, y, z_: Force in x, y, or z-axis
GPa: gigapascals
mm: millimeter
M_x,y,z_: moment in x, y, or z-axis
N: Newtons
N-cm: Newton-centimeters
PEEK: polyether-ether-ketone
PLA: polyactic acid
RPM: rate per minute
SLA: stereolithography
UV: ultraviolet
